# Expanding the Understanding of Biases in Development of Clinical-Grade Molecular Signatures: A Case Study in Acute Respiratory Viral Infections

**DOI:** 10.1371/journal.pone.0020662

**Published:** 2011-06-01

**Authors:** Nikita I. Lytkin, Lauren McVoy, Jörn-Hendrik Weitkamp, Constantin F. Aliferis, Alexander Statnikov

**Affiliations:** 1 Center for Health Informatics and Bioinformatics, New York University School of Medicine, New York, New York, United States of America; 2 Department of Pathology, New York University School of Medicine, New York, New York, United States of America; 3 Division of Neonatology, Department of Pediatrics, Vanderbilt University School of Medicine and Monroe Carell Jr. Children's Hospital at Vanderbilt, Nashville, Tennessee, United States of America; 4 Department of Biostatistics, Vanderbilt University, Nashville, Tennessee, United States of America; 5 Department of Medicine, New York University School of Medicine, New York, New York, United States of America; Dana-Farber Cancer Institute, United States of America

## Abstract

**Background:**

The promise of modern personalized medicine is to use molecular and clinical information to better diagnose, manage, and treat disease, on an individual patient basis. These functions are predominantly enabled by molecular signatures, which are computational models for predicting phenotypes and other responses of interest from high-throughput assay data. Data-analytics is a central component of molecular signature development and can jeopardize the entire process if conducted incorrectly. While exploratory data analysis may tolerate suboptimal protocols, clinical-grade molecular signatures are subject to vastly stricter requirements. Closing the gap between standards for exploratory versus clinically successful molecular signatures entails a thorough understanding of possible biases in the data analysis phase and developing strategies to avoid them.

**Methodology and Principal Findings:**

Using a recently introduced data-analytic protocol as a case study, we provide an in-depth examination of the poorly studied biases of the data-analytic protocols related to signature multiplicity, biomarker redundancy, data preprocessing, and validation of signature reproducibility. The methodology and results presented in this work are aimed at expanding the understanding of these data-analytic biases that affect development of clinically robust molecular signatures.

**Conclusions and Significance:**

Several recommendations follow from the current study. First, all molecular signatures of a phenotype should be extracted to the extent possible, in order to provide comprehensive and accurate grounds for understanding disease pathogenesis. Second, redundant genes should generally be removed from final signatures to facilitate reproducibility and decrease manufacturing costs. Third, data preprocessing procedures should be designed so as not to bias biomarker selection. Finally, molecular signatures developed and applied on different phenotypes and populations of patients should be treated with great caution.

## Introduction

The promise of personalized medicine is to use molecular and clinical information to better diagnose, manage, and treat disease on an individual patient basis. These functions are predominantly enabled by *molecular signatures* that are computational models for predicting phenotypes and other responses of interest from high-throughput assay data. Many molecular signatures have been developed to date from high-throughput data, and some of them have passed regulatory approval and are currently used in clinical practice [1], [2]. However, data-analytics for development of clinically robust molecular signatures is challenging and can undermine the entire effort, if it is not conducted correctly [3]. Whereas substantial tolerance to suboptimal data-analytic protocols (e.g., not perfectly unbiased, slightly underpowered, leading to redundant biomarkers, etc.) exists for exploratory research, extra care has to be taken for development of molecular signatures for clinical use. *Clinical-grade* molecular signatures are subject to vastly more stringent operating quality requirements since such signatures may guide life-and-death decisions. Clinical-grade signatures must also satisfy higher cost-effectiveness and accessibility requirements. In addition, succumbing to data analysis biases can prevent otherwise promising molecular signatures from reaching the market by not meeting requirements for the regulatory approval. In short, design of data-analytic protocols for development of clinical-grade molecular signatures is a very important problem with characteristics distinctively different from those of exploratory data-analytics.

Closing the gap between standards for exploratory versus clinically successful molecular signatures entails a thorough understanding of possible biases in the data analysis phase and developing strategies to avoid them. Previous research has identified several biases of data-analytics for molecular signature development which include: using unsupervised methods (e.g., clustering) for development of molecular signatures [4]; biasing signature accuracy estimation by conducting supervised gene selection both on training and testing data [4], [5]; biasing selection of biomarkers by inappropriately using clinical covariates [6]; and failing to identify predictive signal by using underpowered data-analytic protocols [7] or conducting gene selection for a different phenotype [8].

In the present work we aim to expand the understanding of data-analytic biases that critically affect development of clinically robust molecular signatures. As a case study, we use a recently introduced data-analytic protocol that led to development of a 30-gene “acute respiratory viral response” molecular signature for distinguishing individuals with symptomatic acute respiratory viral infections from uninfected individuals [9]. In a preliminary work we briefly mentioned possible biases of the prior data-analytic protocol related to estimation of signature predictive accuracy, validation of signature in independent data, biomarker redundancy, and signature multiplicity [10]. Here we provide an in-depth technical treatment of these and other biases with an emphasis on what created them and how to avoid them in similar future research. We demonstrate our findings using three datasets that have been recently used for development of molecular signatures of infectious diseases [9], [11], [12]. The conclusions of the present study extend well beyond the development of gene expression-based molecular signature of acute respiratory viral infections; the results readily generalize to other protocols, phenotypes, and assay platforms.

## Materials and Methods

### Microarray gene expression datasets

As the main dataset for development of molecular signatures in this work we used the microarray gene expression dataset of Zaas *et al.* [9] that was downloaded from the Gene Expression Omnibus (GEO) under the accession number GSE17156. This dataset contained 113 normalized gene expression profiles of peripheral blood samples collected from subjects at two time points: (i) prior to inoculation with one of three respiratory viruses (HRV, RSV and influenza A) and (ii) at the peak time of symptoms. The pre-inoculation samples are referred to as *baseline* or *unexposed* samples. The post-inoculation samples are referred to as *peak time* or *exposed*. One of the 113 samples (GSM429232) did not have a matching baseline gene expression profile and was excluded from analysis. Thus, the dataset used in this work contained in total 112 gene expression profiles. Their break down by virus type and time of collection (baseline or peak) is shown in Table 1. All subjects were healthy and uninfected at baseline with some remaining asymptomatic after the viral exposure, while others developed symptoms of a viral infection as shown in Table 1. Exposed subjects were considered asymptomatic if their modified Jackson score [13] was below 6 over the 5 days of observation and if the viral shedding was not detected after the first 24 hours post inoculation [9]. Thus, following Zaas *et al.* [9], we also consider asymptomatic subjects to be uninfected.

**Table 1:** Number of gene expression profiles corresponding to each category of samples from the data of Zaas *et al.* [9] and Ramilo *et al.* [12].

*Infection type*	*Zaas et al.*		*Ramilo et al.*
	*Asymptomatic*	*Symptomatic*	
**Rhinovirus** (HRV)	10	9	N/A
**Respiratory Syncytial Virus** (RSV)	11	9	N/A
**Influenza A**	9	8	18
**Bacterial** (*Staphylococcus aureus*, *Streptococcus pneumoniae*, *Escherichia coli*)	N/A	N/A	73
**Unexposed** (healthy uninfected)	56		6

The dataset of Ramilo *et al.* [12] was used for an independent validation of panviral molecular signatures developed in the present work and was also obtained from GEO (accession number GSE6269). This dataset contained gene expression profiles obtained from peripheral blood leukocytes of mostly pediatric patients with acute infections caused by either influenza A, or one of three bacterial pathogens: (i) *Staphylococcus aureus*, (ii) *Streptococcus pneumoniae*, both Gram-positive bacteria, and (iii) *Escherichia coli*, a Gram-negative bacterium. The data of Ramilo *et al.* [12] also contained gene expression profiles of 6 healthy controls. Distribution of the number of gene expression profiles for each group of patients is shown in Table 1.

Finally, a third dataset was used to demonstrate that the overall conclusions of the present paper pertaining to data-analytic protocols, generalize beyond the domain of acute respiratory viral infections. This dataset originated from a recent study aimed at development of molecular signatures for diagnosis of invasive Candidemia, one of the most common bloodstream infections in the U.S. [11]. The dataset contained 72 normalized gene expression profiles of peripheral blood samples from mice and was downloaded from GEO (accession number GSE20524). Out of 72 samples, 46 were infected with *C. albicans*, 9 were infected with *S. aureus* bacteremia (the most common bloodstream infection occurring in patients at risk for Candidemia), and 17 were healthy controls.

### Simulated data used for evaluation of methods for development of molecular signatures under the condition of signature multiplicity

In order to compare, in a controlled setting, methods for developing molecular signatures considered in this work, we use a simulated dataset *TIED* with exactly known causal relationships between variables [14], [15] and which was previously used in an international causality challenge [16]. The data generating graph is shown in Figure S1 and its parameterization is provided in [14], [15]. The dataset contains 750 observations and 1,000 variables (999 genes and a phenotypic response variable). There are 72 distinct molecular signatures of the phenotype (i.e., sets of non-redundant genes that carry maximal predictive information about the phenotype and render it statistically independent of all other genes). Each of these signatures carries equivalent information about the phenotype and spans over 5 genes: gene *X*_10_ and one gene from each of the four subsets {*X*_1_,*X*_2_,*X*_3_,*X*_11_}, {*X*_5_,*X*_9_}, {*X*_12_,*X*_13_,*X*_14_} and {*X*_19_,*X*_20_,*X*_21_}.

### Method for developing multiple molecular signatures of the same phenotype

A perplexing phenomenon that characterizes high-throughput data analysis is the ubiquitous multiplicity of molecular signatures [17], [18]. This phenomenon has far-reaching implications for biological discovery and development of next generation patient diagnostics and personalized treatments [15]. Therefore, it is informative not only to show the existence of a single signature for a given phenotype variable, but also to seek all possible maximally predictive signatures that do not contain redundant genes. Such analysis allows to improve discovery of the underlying biological mechanisms by not missing genes that are implicated mechanistically in the disease processes. Furthermore this analysis facilitates separation of statistical instability from intrinsic information equivalency [15].

To extract multiple molecular signatures, we apply a recently introduced and provably correct algorithm TIE* that outputs the complete set of maximally predictive and non-redundant signatures independent of the data distribution [15]. TIE* is based on Markov boundary induction which enables probabilistic modeling of multiple signatures and formally connects them with the causal graph (pathway) of the data generating process even when this pathway is not known a priori [19]–[22]. TIE* has been shown to have excellent sample and computational efficiency and to extract signatures reproducible in independent datasets [15].

In this work, we use Generalized Local Learning (abbreviated as GLL; specific instantiation: semi-interleaved HITON-PC without symmetry correction) as the base Markov boundary algorithm in TIE* [23], [24]. This choice of the base algorithm was motivated by its empirical performance in microarray gene expression and other high-throughput data as well as its theoretical properties [23], [24]. Under broad assumptions, GLL provably discovers non-redundant genes that are located in the local pathway of the phenotype variable [23], [24]. GLL was run with the Fisher's *Z*-test for vanishing partial correlations at significance level 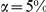, and with *max-k* = 1. The maximum cardinality of a subset of genes to be excluded from the entire set of genes within each iteration in TIE* was set to 5. Fisher's *Z*-test was also used for evaluation of candidate Markov boundaries in TIE* [15].

Once genes were selected, we completed the development of molecular signatures by applying Support Vector Machine (SVM) classifiers [25] implemented in LibSVM version 2.89 (http://www.csie.ntu.edu.tw/~cjlin/libsvm). SVMs were applied with the linear kernel and the cost parameter *C* = 100.

### Method for assessing redundancy of genes in a molecular signature

A gene is considered to be redundant with respect to the phenotype if its removal from the molecular signature does not decrease the signature's predictive accuracy. Thus, in principle, redundancy can be assessed using so-called wrapper algorithms [26]. However, wrapping techniques are prone to overfitting due to a very large number of comparisons, and in small-sample settings they can falsely conclude that different sets of biomarkers have the same predictive accuracy when in reality they do not [23]. Thus we test the redundancy of genes using a more conservative approach with the following two steps. First, we find genes that do not carry any association with the phenotype conditioned on another gene from the signature using Fisher's Z-test [27] at significance level 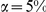. Once we identify such genes, we do not readily exclude them from the molecular signature but do so only if their removal does not lead to decrease in predictive accuracy of the signature (as measured by the area under ROC curve and compared using statistical test of Delong *et al.* [28]). The assessment of redundancy is performed by repeated cross-validation [29] in training data only. The resulting non-redundant signature is subsequently validated in an independent data and its predictive accuracy is compared to the accuracy of the original signature (which contains both redundant and non-redundant genes).

### Method for assessing biases of data preprocessing after standard microarray data normalization

In order to study the effects on gene selection of different data preprocessing schemes (discussed below) following the standard microarray data normalization (e.g., by the RMA method [30], [31]), we employ permutation testing with 10,000 permutations of the phenotype variable under the null hypothesis of no association between genes and the phenotype. On each permutation, we apply a given data preprocessing method and then perform gene selection using a two-sample *t*-test with the false discovery rate (FDR) correction at level 0.2 [32], [33]. This procedure allows us to quantify the extent to which different preprocessing methods may bias gene selection. Under the assumption that the null hypothesis holds in the data and if a preprocessing method does not bias gene selection, we would expect none of the genes to be selected as significantly associated with the phenotype. However, since we are simulating the null hypothesis by permuting the phenotype variable in a real dataset where expression of different genes may not be independent of others and where small sample effects may be present, a small number of genes may be deemed significantly associated with the phenotype even under an unbiased preprocessing method. Because such effects are expected to be minimal when no preprocessing is performed, we use it as a baseline against which all other preprocessing methods can be evaluated. If after some preprocessing, the number of significantly associated genes increases relative to no preprocessing, this signals that the applied preprocessing method may bias gene selection by potentially increasing the number of false positives.

### Data preprocessing methods

We consider six different data preprocessing methods that are applied in the current study after the standard microarray data normalization by RMA. The first method establishes a baseline and consists of no preprocessing. The second method was used in the protocol of Zaas *et al.* [9] and consists of centering the data by subtracting the grand mean from the entire gene expression dataset. The third method standardizes each gene expression variable to have zero mean and standard deviation of one. The fourth method rescales each gene expression variable to lie in the interval [0,1]. These four methods are commonly used in gene expression analysis and are unsupervised in a sense that they do not take into account the phenotype information, and thus are unlikely to introduce gene selection biases.

The fifth preprocessing method considered here was implemented in the supplementary software of Zaas *et al.* [9] and was aimed at correcting differences between gene expression profiles of the uninfected subjects from different experimental cohorts (i.e., HRV, RSV and Influenza A). This correction was performed using all subjects within each cohort by first computing the mean gene expression profile of the uninfected subjects within the cohort and then subtracting this mean profile from all gene expression profiles (i.e., infected and uninfected) in the cohort. The key assumptions underlying this preprocessing method are that all uninfected subjects should have similar gene expression profiles and that the observed differences are entirely due to the so-called “batching effect” arising from technical variation when assaying biological samples. An illustration of the effects of this preprocessing method is given in the Text S1.

The sixth preprocessing method considered here is ComBat [34], which also aims to alleviate the influence of batch effects on the analysis of gene expression data [35]. ComBat relies on two assumptions: (i) that all uninfected subjects should have similar gene expression profiles and (ii) that batch effects affect gene expression measurements in a similar way across many genes. In ComBat, batch effects are first modeled as additive (i.e., location) and multiplicative (i.e., scale) components of the observed gene expression levels for each gene. These estimates are then updated in a Bayesian framework that pools information on batch effect estimates from all the genes in the dataset. We chose ComBat for our evaluation due to this method's computational efficiency and lack of ad-hoc parameters, which makes ComBat appropriate for application in a permutation-based framework. When applying ComBat, the phenotype was supplied as a covariate in addition to the cohort incidence variable. This was done in order to allow ComBat to retain the variation in gene expression profiles that was due to biological responses to pathogens.

## Results and Discussion

### A simulation study demonstrating data-analytic biases related to signature multiplicity and biomarker redundancy

Evaluation of data-analytic protocols in real data is challenging due to absence of a biological gold standard describing true interactions between genes and the phenotype. For this reason and in order to illustrate in a controlled environment, the behavior of the factor analysis-based gene selection method from the protocol of Zaas *et al.* [9], we conducted experiments in a simulated dataset *TIED* with exactly known causal relationships between variables [14], [15]. This dataset allows us to evaluate the considered data-analytic protocol in terms of its effectiveness in extracting the complete set of relationships between genes and the phenotype. Identification of these relationships is essential for constructing a comprehensive view of the underlying biological process.

When applied to *TIED*, factor analysis-based method extracted only a single signature containing 30 genes, 4 of which were causally relevant and non-redundant (*X*_5_, *X*_10_, *X*_12_ and *X*_20_), 4 were redundant given the previous set of genes (*X*_8_, *X*_13_, *X*_14_ and *X*_21_), and 22 were irrelevant and without association with the phenotype. (see Figure S1 for an illustration of the complete data-generating graph of causal relationships that produced *TIED* dataset). In particular, the factor analysis-based technique missed all genes from the subset {*X*_1_,*X*_2_,*X*_3_,*X*_11_} that are causally directly related to the phenotype. In contrast, TIE* correctly identified all and only the 72 non-redundant molecular signatures of the phenotype in *TIED* dataset. These results indicate that the factor analysis-based protocol leads to selection of false positive and false negative genes.

### There exist many different and equally accurate molecular signatures of the panviral phenotype

Using the TIE* algorithm, we identified 3,473 novel non-redundant and maximally predictive signatures of acute respiratory viral infections in the dataset of Zaas *et al.*, while the prior data-analytic protocol yielded only one signature of the phenotype [9]. On average each identified novel signature contained 11 genes, and together all signatures spanned over 60 distinct oligonucleotide probes corresponding to 57 genes. The average phenotype classification performance of these signatures in the independent data of Ramilo *et al.* [12] was 0.92 area under the ROC curve (AUC) with a standard deviation of 0.06 AUC. Notably, 3,308 (or 95%) of the signatures discovered by TIE* achieved classification performance comparable to the panviral signature of Zaas *et al.* Genes that appeared in more than 20% of the signatures are shown in Table 2. Out of these genes, only three genes (*RSAD2*, *IFI44L* and *IFI44*) were present in the panviral signature of Zaas *et al.* In contrast, all 12 genes comprising the panviral signature that we previously developed [10] were among genes listed in Table 2 (highlighted in bold). The complete list of molecular signatures discovered by TIE* and the genes comprising those signatures can be found in the Dataset S1 and Table S1, respectively.

**Table 2:** Genes that appeared in more than 20% of non-redundant and maximally predictive signatures identified by TIE* for discriminating between symptomatic and uninfected samples.

*Probe ID*	*Gene symbol*	*Gene name*	*Percentage of signatures participated in*
201065_s_at	***GTF2I***	general transcription factor IIi	73%
213674_x_at	***IGHD***	immunoglobulin heavy constant delta	73%
214511_x_at	***FCGR1B***	Fc fragment of IgG, high affinity Ib, receptor (CD64)	72%
207826_s_at	***ID3***	inhibitor of DNA binding 3, dominant negative helix-loop-helix protein	71%
213797_at	***RSAD2***	radical S-adenosyl methionine domain containing 2	71%
217418_x_at	***MS4A1***	membrane-spanning 4-domains, subfamily A, member 1	70%
219471_at	***C13orf18***	chromosome 13 open reading frame 18	69%
219112_at	***RAPGEF6***	Rap guanine nucleotide exchange factor (GEF) 6	63%
219073_s_at	***OSBPL10***	oxysterol binding protein-like 10	59%
219313_at	***GRAMD1C***	GRAM domain containing 1C	56%
204439_at	***IFI44L***	interferon-induced protein 44-like	42%
221234_s_at	*BACH2*	BTB and CNC homology 1, basic leucine zipper transcription factor 2	29%
216950_s_at	*FCGR1A*, *FCGR1C*	Fc fragment of IgG, high affinity Ia, receptor (CD64); Fc fragment of IgG, high affinity Ic, receptor (CD64)	28%
207431_s_at	***DEGS1***	degenerative spermatocyte homolog 1, lipid desaturase (Drosophila)	25%
205049_s_at	*CD79A*	CD79a molecule, immunoglobulin-associated alpha	24%
202723_s_at	*FOXO1*	forkhead box O1	22%
44790_s_at	*C13orf18*	chromosome 13 open reading frame 18	22%
203413_at	*NELL2*	NEL-like 2 (chicken)	20%
214059_at	*IFI44*	Interferon-induced protein 44	20%

Genes highlighted in bold are those that also comprised the 12-gene panviral signature developed by applying GLL on the entire set of samples [10].

Since the phenotype is characterized by *multiple* molecular signatures, focusing on a *single* arbitrarily chosen signature may not yield causative biomarkers of the disease nor provide accurate grounds for understanding pathogenesis [15]. In general, genes comprising a single molecular signature may not be the only determinants of the phenotype. There may exist multiple equally informative and non-redundant gene sets that when taken together would provide a comprehensive view of the underlying biological process. Therefore, data-analytic protocols should extract, to the extent possible, *all* molecular signatures of the phenotype.

### Many genes in the previously developed “acute respiratory viral response” signature are redundant

Our redundancy analysis showed that only 20 gene probes from the 30-gene panviral signature (corresponding to 32 gene probes) identified by the factor analysis-based gene selection method from the data-analytic protocol of Zaas *et al.* [9] were non-redundant. The following gene probes were found to be redundant (gene names are provided in parentheses): 202672_s_at (*ATF3*), 218943_s_at (*DDX58*), 219863_at (*HERC5*), 214059_at (*IFI44*), 214453_s_at (*IFI44*), 204439_at (*IFI44L*), 204415_at (*IFI6*), 204747_at (*IFIT3*), 205483_s_at (*ISG15*), 205569_at (*LAMP3*), 202145_at (*LY6E*), and 202086_at (*MX1*). A panviral signature constructed on the basis of the 20 remaining non-redundant genes achieved the same predictive accuracy (1.0 AUC) in the independent validation data of Ramilo *et al.* [12] as the original signature.

It should be noted, however, that redundancy is not always equivalent to biological irrelevance of the genes, but only implies that the redundant genes do not carry any additional predictive information about the phenotype beyond what's conveyed by the non-redundant genes. While presence of redundant genes in a signature could potentially worsen its reproducibility and would surely increase its manufacturing costs, in some cases it may be desirable to explicitly engineer redundancy into a molecular signature in order to improve its robustness for a specific phenotype. We would argue, however, that such redundancy should arise from a careful methodological design rather than being an unintended consequence of applying a certain data analytic technique.

### Data preprocessing methods may bias gene selection

While the topic of microarray gene expression data normalization has been extensively studied in prior work [31], [36], the effects on gene selection of data preprocessing after normalization remain unclear. Below, we present a comparison of six different data preprocessing methods using gene expression dataset of Zaas *et al.* [9]. Since it is not known which genes are truly associated with the panviral phenotype, we conducted phenotype label permutation experiments under the null hypothesis of no association between genes and the phenotype. The results are shown in Table 3 and demonstrate that no preprocessing, centering, standardization and [0,1] scaling did not bias gene selection. The average number of significantly associated genes under each of these preprocessing methods was 0.3 with standard error 0.091 over 10,000 permutations. However, the batch correcting procedure from the supplementary software of Zaas *et al.* biased gene selection and resulted in an average of 55.6 genes being deemed significant with standard error 4.407. Similarly, application of the ComBat batch correction method produced 71.3 (standard error 5.051) significant genes on average.

**Table 3:** Effects of preprocessing methods on gene selection under the null hypothesis of no association between genes and the panviral phenotype in the acute respiratory viral infections dataset [9].

*Preprocessing*	*Number of significant probes*			
	*Mean*	*St. Dev.*	*95% Interval*	
No preprocessing	0.3	9.1	0.0	0.0
Center (subtract global mean)	0.3	9.1	0.0	0.0
Standardize (subtract global mean and divide by stdev)	0.3	9.1	0.0	0.0
Scale each probe to [0,1]	0.3	9.1	0.0	0.0
Batch correction from the supplementary software of Zaas et al. [9]	55.6	440.7	0.0	287.5
ComBat	71.3	505.1	0.0	707.0

The phenotype variable was randomly permuted 10,000 times. On each permutation, we applied a given preprocessing method and then performed gene selection using a two-sample *t*-test with the false discovery rate (FDR) correction at level 0.2 [32], [33].

We note that these results were obtained in simulated conditions of no biological signal in the data. A study of behavior of the considered batch correction methods under the alternative hypothesis of presence of a biologically meaningful signal remains an open research direction that would have to rely on biological validation of genes selected on preprocessed data. Preliminary results reported here suggest that the two batch correction methods may potentially lead to an increase in the number of false positives in the output of statistical methods for gene selection. We hypothesize that the increased number of statistically significant genes came as a result of decreases in within-group variance in expression of genes in the infected and uninfected groups of subjects after correcting for batch effects. When not offset by a comparable decrease in differences between the groups' mean gene expression profiles, this decrease in variance may cause an appearance of statistically significant associations between the phenotype and genes that were not significantly differentially expressed before data preprocessing. We further illustrate this behavior in Figure S2 that shows distributions of variance of gene expression in the original (i.e., non-permuted) infected and uninfected subjects as well as differences between mean expression of genes in the two groups of subjects before and after preprocessing by the supplementary software of Zaas *et al.* As made evident by Figure S2, preprocessing reduced within-group variances of gene expression while leaving differences between group means largely unchanged. Data preprocessing by ComBat had a very similar effect and the corresponding histograms are shown in Figure S3.

**Figure 1:**
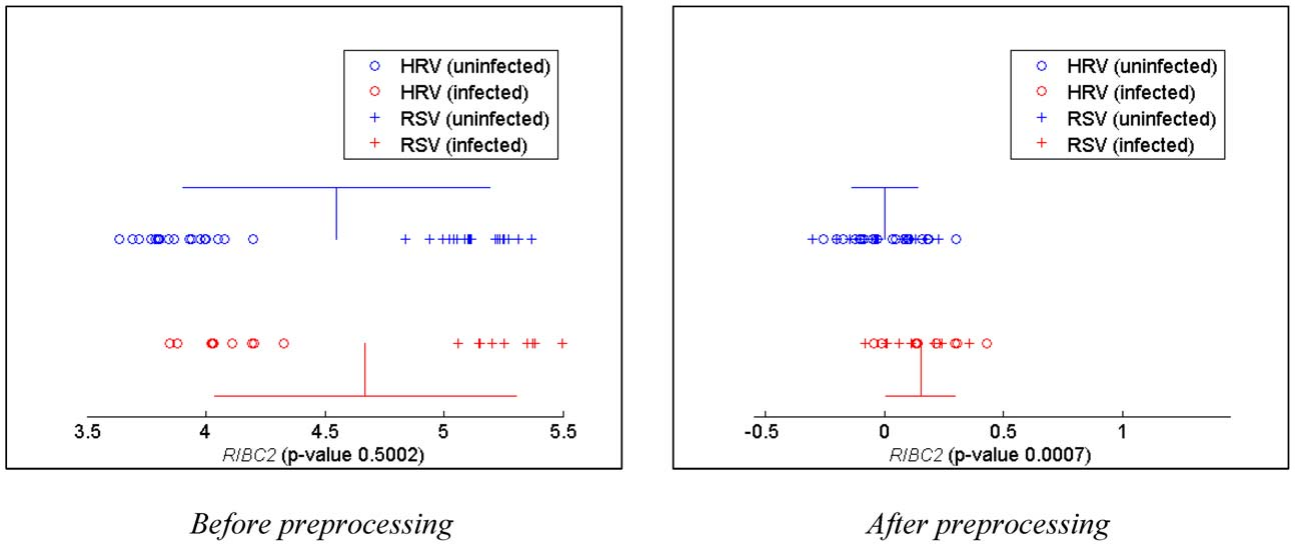
Effects of preprocessing by the supplementary software of Zaas *et al.* **[9]** on real gene expression data. Gene expression profiles of the uninfected subjects are shown in blue staggered on top of the profiles of the infected subjects highlighted with red. The blue and red vertical line segments denote locations of the mean expression in the uninfected and infected groups, respectively. Likewise, blue and red horizontal line segments emanating in both directions from the means denote one standard deviation within the uninfected and infected groups, respectively. P-values produced by a two-sample *t*-test with unequal variances are shown in parenthesis.

A specific example illustrating the above effects of preprocessing in the original (non-permuted) data is shown in Figure 1. As can be seen in that figure, within-class variances decreased roughly five-fold for gene *RIBC2* as a result of batch correction using the supplementary software of Zaas *et al*. Consequently, the p-value produced by a two-sample *t*-test for differential expression decreased from roughly 0.5 to below 10^−3^ causing an appearance of a statistically significant association between gene *RIBC2* and the panviral phenotype. Although the two classes of gene expression profiles could not be separated without errors using only gene *RIBC2* in the preprocessed data, in general, such preprocessing may force classes to become perfectly separable as shown using simulated data in Figure S4.

Similar effects of preprocessing were observed in a large portion of genes in the data of Zaas *et al.* [9]. We applied a two-sample *t*-test with FDR 0.2 [33] to identify genes statistically significantly associated with the panviral phenotype, either before or after preprocessing. While only 1,759 genes were significantly associated with the phenotype in the original data, the number of significant genes in the preprocessed data was four times higher, amounting to 7,347 genes when the data was preprocessed using the supplementary software of Zaas *et al.* and 7,557 genes when using ComBat. Notably, all genes that were significantly associated with the phenotype before preprocessing remained significant after preprocessing. Therefore, none of the genes lost their association and 5,588 (5,798 for ComBat) genes gained association with the phenotype as a result of preprocessing.

These findings indicate that special care has to be taken when applying preprocessing methods for gene expression analysis. Batch effect correction methods may be appropriate for application in cases when significant biological differences between samples can be ruled out. However, the cohort recruitment protocol of Zaas *et al.* does not allow such biological differences to be ruled out without additional validation. According to Zaas *et al.*, the HRV cohort was recruited through an active screening protocol at the University of Virginia, so these subjects may be a younger, healthier group mostly composed of college students, and are more likely to be middle to upper-middle class. The RSV cohort was recruited and infected through Retroscreen Virology, London, a company that specializes in clinical trials on viruses. It is likely that the ages of the subjects are more diverse than the HRV cohort, and perhaps the racial make-up is more diverse as well since London has a more ethnically diverse population than Charlottesville, Virginia. The influenza cohort was recruited and infected through Retroscreen Virology, Brentwood, UK. Brentwood is 20 miles outside of London, a suburban setting. It is likely that volunteers are more diverse in age and less racially diverse than the London (RSV) cohort.

Given the above stated observations on the effects of batch correction methods and due to a lack of information regarding the causes of differences between the unexposed samples from different viral cohorts in the data of Zaas *et al.* [9], we used only the RMA normalization in our data analysis.

### Molecular signatures should be developed and applied to the same phenotype and population of subjects

The 30-gene panviral molecular signature introduced by Zaas *et al.* [9] was developed specifically for differentiating between uninfected (healthy) subjects and subjects who developed symptoms following a viral inoculation with either HRV, RSV, or influenza A. In an attempt to demonstrate specificity of this molecular signature to viral infections, Zaas *et al.* applied this signature for classification of subjects with bacterial and viral (Influenza A) infections in the data of Ramilo *et al.* [12] and reported a predictive accuracy of roughly 0.94 AUC [9]. This interesting result raises the following question that we address below: Why a molecular signature developed for one task (*differentiating between uninfected subjects and subjects with viral infections*) was successful in performing another task (*differentiating between subjects with bacterial and viral infections*)?

**Figure 2:**
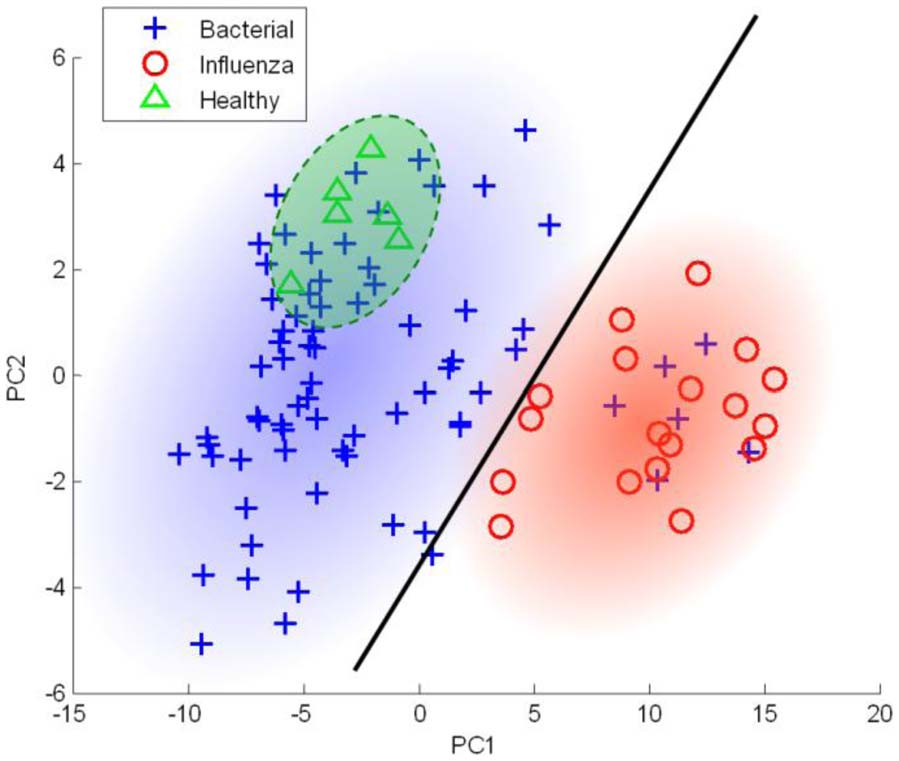
Visualization of subjects in the dataset from **[12]** in the space of the first two principal components of the panviral signature of Zaas *et al.* The solid line is an approximation of the molecular signature (classifier) of Zaas *et al.*; subjects to the left of this line are classified as uninfected (healthy) and subjects to the right are classified as virally infected (Influenza A). Blue and red gradient highlighting corresponds to the regions where the majority of bacterial and viral profiles belong, respectively. Green highlighting shows the area with uninfected (healthy) profiles.

Figure 2 graphically depicts subjects from the dataset of Ramilo *et al.* in the space of the first two principal components obtained from genes that constituted the panviral signature of Zaas *et al.* The solid line is an approximation of the molecular signature (classifier) of Zaas *et al.* This signature would classify subjects to the left of the line as uninfected (healthy) whereas subjects to the right of the line would be classified as virally infected. Figure 2 also demonstrates that the same molecular signature can incidentally be used to accurately differentiate between subjects with bacterial and viral infections from the dataset of Ramilo *et al.*, thus confirming the finding of Zaas *et al.* However, this result is due to a lucky choice of genes in the molecular signature of Zaas *et al.* that was either helped by redundant genes for the viral phenotype (recall that only 20 gene probes were non-redundant) and/or could have been informed by other criteria and procedures not reported in the original publication. When we substituted factor analysis-based gene selection in the protocol of Zaas *et al.* with GLL, which by design yields only non-redundant genes for the viral phenotype, predictive accuracy for the bacterial vs. viral classification task was reduced to 0.60 AUC. This indicates that the finding of Zaas *et al.* is method-dependent. Moreover, the following subsection shows that the methodology employed by Zaas *et al.* for evaluating the specificity of their molecular signature to viral infections does not generalize to other datasets.

These results demonstrate that molecular signatures developed for one phenotype and population of subjects and applied to another phenotype and/or population are highly problematic. There is no reason to undertake this risk when one can apply supervised techniques to data for the *same* phenotype and population of subjects. Specifically, in case of performing classification of virally and bacterially infected subjects, one would need to develop a new molecular signature using gene expression profiles of patients with viral and bacterial infections. Although this recommendation may seem obvious, current practices in clinical research suggest otherwise. For instance, extrapolation of results obtained using animal models to humans has been a de-facto methodology underlying much of translational clinical and biomedical research. However, animal models are often not representative of the effects an intervention may have in humans [37]–[39]. Therefore, in cases when applications of a model in a different organism or phenotype cannot be justified biologically, data-analytic protocols should be applied to construct organism- and phenotype-specific models.

### Conclusions of this case study generalize beyond the domain of acute respiratory viral infections

Below we demonstrate that the major findings of this case study pertaining to data analytic protocols generalize to other domains and datasets. We analyze a microarray gene expression dataset that was used for development of molecular signatures for diagnosis of Candidemia [11]. We chose this dataset because it has been previously analyzed with a protocol that is very similar to the one applied for development of acute respiratory viral infection signatures [9]. Note that since the Candidemia dataset was collected from a different organism than the acute respiratory viral response dataset and because Candidemia is a drastically different disease than respiratory infections, we do not draw comparisons between genes comprising molecular signatures of Candidemia and genes comprising molecular signatures of the panviral phenotype discussed earlier. In what follows, we only compare molecular signatures developed in the Candidemia dataset.

By employing factor analysis, the original study showed the existence of a single 82-gene signature that accurately classified Candidemia-infected samples versus healthy controls [11]. Using the TIE* algorithm, we identified 2,922 novel non-redundant and maximally predictive signatures of Candidemia in the same set of training samples. On average, each novel signature contained 14 genes, and together all signatures spanned over 65 distinct genes. The average phenotype classification performance of these signatures in the testing set of samples was 0.996 AUC with a standard deviation of 0.01 AUC. Notably, 2,513 (or 86%) of the signatures discovered by TIE* achieved AUC = 1.0. The complete list of molecular signatures discovered by TIE* and the genes comprising those signatures can be found in Dataset S2 and Table S2, respectively. Interestingly, there were no genes in common between the Candidemia signature of Zaas *et al.* [11] and multiple signatures indentified by TIE*.

We have further assessed redundancy within the 82-gene signature and found 79 redundant and only 3 non-redundant gene probes: 1449453_at (*Bst1*), 1424254_at (*Ifitm1*), 1421304_at (*Klra2*). A molecular signature developed on the basis of these three non-redundant genes achieved predictive accuracy of 1.0 AUC in the independent validation data, which is the same as the accuracy of the original 82-gene signature.

Next, we observed that the effects of batch correction methods on gene selection extend beyond the acute respiratory viral infections dataset and that such preprocessing also biases gene selection in the Candidemia dataset. In this case, there were two experimental batches corresponding to samples from the *C. albicans* and *S. aureus* cohorts, respectively. Each batch contained samples from infected and uninfected mice. The phenotype variable differentiated between infected and uninfected samples. Experiments conducted under the null hypothesis of no association between the genes and the phenotype produced results consistent with the ones obtained in the acute respiratory viral infections dataset. As can be seen in Table 4, the average number of significantly associated genes under no preprocessing, centering, standardization and [0,1] scaling was 82.6 with standard error 6.407 over 10,000 permutations. The number of significantly associated genes increased to an average of 221.8 with standard error 10.98 when preprocessing from the supplementary software of Zaas *et al.* was applied. Similarly, preprocessing by ComBat resulted in 253.2 (standard error 11.743) significant genes on average.

**Table 4:** Effects of preprocessing methods on gene selection under the null hypothesis of no association between genes and the bacterial phenotype in the Candidemia dataset [11].

*Preprocessing*	*Number of significant probes*			
	*Mean*	*St. Dev.*	*95% Interval*	
No preprocessing	82.6	640.7	0.0	607.0
Center (subtract global mean)	82.6	640.7	0.0	607.0
Standardize (subtract global mean and divide by stdev)	82.6	640.7	0.0	607.0
Scale each probe to [0,1]	82.6	640.7	0.0	607.0
Batch correction from the supplementary software of Zaas et al. [9]	221.8	1098.0	0.0	3543.5
ComBat	253.2	1174.3	0.0	3991.5

The phenotype variable was randomly permuted 10,000 times. On each permutation, we applied a given preprocessing method and then performed gene selection using a two-sample *t*-test with the false discovery rate (FDR) correction at level 0.2 [32], [33].

Application of the two-sample *t*-test with FDR 0.2 to the original Candidemia dataset [11] (i.e., raw probe data after RMA normalization) produced 11,256 genes that were significantly associated with the phenotype differentiating between bacterially infected and uninfected samples. However, the same experiment in the data after preprocessing resulted in 13,590 significantly associated genes when using the supplementary software of Zaas *et al.* and 13,850 genes after preprocessing with ComBat. Similarly to the results obtained in the acute respiratory viral infections dataset, none of the genes in the Candidemia dataset lost their association and 2,334 and 2,594 genes gained association with the phenotype as a result of preprocessing with the two batch correction methods.

Finally, we applied the 82-gene molecular signature of Candidemia to classify *S. aureus* bacteremia and *C. albicans* in the independent set of 27 samples (18 *C. albicans* and 9 *S. aureus*) [11]. This experiment was designed to mimic the signature specificity validation step from the original data-analytic protocol of Zaas *et al.* [9]. In this case, however, performance of the Candidemia signature did not generalize to the different phenotype, resulting in classification accuracy statistically indistinguishable from that of a signature with no predictive power (0.5 AUC). In addition, the study [11] has reported the ability to accurately classify the two bloodstream infections using a *new* molecular signature that was specifically designed for that classification task. Taken together with the results of our analysis, this further accentuates the need to develop and apply molecular signatures to the same phenotype and population of subjects.

### Conclusion and operational recommendations

The science and technology of molecular signatures is positioned to play a crucial role in the advancement of personalized medicine and clinical diagnostics. Data-analytics is a central component of molecular signature development. On the basis of many recent meta-analyses and re-analyses of prior experiments it becomes evident that biased data analytics are emerging as a major obstacle for progress in personalized medicine [3]–[7], [40]. Improving data-analytics for development of clinical-grade molecular signatures requires detailed understanding of the data analysis biases and development of strategies to avoid them. In this work, we presented a case study evaluating a data-analytic protocol that has recently led to development of an important 30-gene signature of acute respiratory viral infections [9] and also informed the development of the 82-gene signature of Candidemia [11]. Conclusions of this study, however, are not specific to the analysis of acute respiratory viral infections and generalize to other domains as was made evident by validation of our results in additional data. Below we summarize our key findings and operational recommendations to data analysts based on empirical results reported in this paper.

First, we showed the existence of many different and equally accurate molecular signatures of the phenotypes. Therefore, in order to obtain comprehensive and accurate grounds for understanding pathogenesis, data-analytic protocols should extract, to the extent possible, *all* molecular signatures of a phenotype rather than focusing on an arbitrarily chosen *single* signature. Whenever possible, analysis that separates statistical instability from intrinsic information equivalency should be undertaken [15]. Second, our results demonstrate the presence of redundant genes in prior molecular signatures and highlight the need for routine assessment of molecular signatures for redundancy with respect to the phenotype. Generally, if some genes are found to be redundant, they can be excluded from the molecular signature, because such genes do not contribute additional predictive information about the phenotype and have the potential to worsen signature reproducibility and increase its manufacturing costs. In certain cases, however, it may be necessary to explicitly engineer redundancy into a molecular signature in order to improve its robustness for a specific phenotype. Such redundancy should arise from a careful methodological design rather than being an unintended consequence of applying a certain data analytic technique. Third, we showed that data preprocessing may bias gene selection. It is therefore necessary to assess the effects of any preprocessing and other steps of data-analytic protocols on selection of genes. Furthermore, subsequent analyses should not assume that the same preprocessing would be appropriate in a different setting. Finally, molecular signatures should be developed and applied to the same phenotype and population of subjects. Failure to do so may result in spurious findings and non-reproducible data-analytic protocols, as was demonstrated in the present study. The methodology and results presented in this work combined with previously established bias avoidance strategies aim to further advance the process of development of clinically successful molecular signatures by improving the associated data-analytic protocols.

## Supporting Information

Figure S1 **Data generating graph that was used for evaluation of methods for development of molecular signatures under the condition of signature multiplicity.** There are 1,000 variables in the graph (999 genes and a phenotypic response variable *T*). Genes that contain exactly the same information about *T* are highlighted with the same color, e.g. genes *X*_12_, *X*_13_, and *X*_14_ provide exactly the same information about *T* and are thus interchangeable for prediction of *T*. There are 72 distinct molecular signatures of the phenotype *T* (i.e., sets of non-redundant genes that carry maximal predictive information about the phenotype and render it statistically independent of all other genes). Each of these signatures carries equivalent information about the phenotype and spans over 5 genes: gene *X*_10_ and one gene from each of the four subsets {*X*_1_,*X*_2_,*X*_3_,*X*_11_}, {*X*_5_,*X*_9_}, {*X*_12_,*X*_13_,*X*_14_} and {*X*_19_,*X*_20_,*X*_21_}.
(TIF)

Figure S2 **Distributions of variance in the infected and uninfected subjects (top two figures) and differences between means of their gene expression profiles (bottom) before and after preprocessing by the supplementary software of Zaas** ***et al.*** **** [**9**] **.** The distribution of variance is shifted to the left (i.e., to smaller values) as a result of preprocessing, while the distribution of differences between means is largely unaffected.
(TIF)

Figure S3 **Distributions of variance in the infected and uninfected subjects (top two figures) and differences between means of their gene expression profiles (bottom) before and after preprocessing by ComBat** [**34**] **.** The distribution of variance is shifted to the left (i.e., to smaller values) as a result of preprocessing, while the distribution of differences between means is largely unaffected.
(TIF)

Figure S4 **Effects of preprocessing method from the supplementary software of Zaas** ***et al.*** **** [**9**] **on simulated data.** Gene expression profiles of the uninfected subjects are shown in blue staggered on top of the profiles of the infected subjects highlighted with red. The blue and red vertical line segments denote locations of the mean expression in the uninfected and infected groups, respectively. Likewise, blue and red horizontal line segments emanating in both directions from the means denote one standard deviation within the uninfected and infected groups, respectively. P-values produced by a two-sample *t*-test with unequal variances are shown in parenthesis.
(TIF)

Table S1 The complete list of genes participating in the 3,473 non-redundant and maximally predictive molecular signatures discovered by the TIE* algorithm in the data of Zaas *et al.* [9] for discriminating symptomatic from uninfected samples. Genes highlighted in bold are those that also comprised the 12-gene panviral signature developed by Statnikov *et al.* [10] by applying GLL on the entire set of samples.
(PDF)

Table S2 The complete list of genes participating in the 2,922 non-redundant and maximally predictive molecular signatures discovered by TIE* in the data of Zaas *et al.* [11] for discriminating between Candidemia-infected samples and healthy controls. Genes highlighted in bold are those that also comprised a 14-gene signature developed by applying GLL in the same data.
(PDF)

Dataset S1 The complete list of molecular signatures discovered by TIE* for the panviral phenotype from the data of Zaas *et al.* [9].
(CSV)

Dataset S2 The complete list of molecular signatures of Candidemia discovered by TIE* in the data of Zaas *et al.* [11].
(CSV)

Text S1 An illustration of the effects of a preprocessing procedure from the supplementary software of Zaas *et al.* [9].
(PDF)
