# Supplementary figures and images for: Expanding the Understanding of Biases in Development of Clinical-Grade Molecular Signatures: A Case Study in Acute Respiratory Viral Infections
Source: PLoS One. 2011 Jun 1;6(6):e20662. doi: 10.1371/journal.pone.0020662 (PMC3105991; doi:10.1371/journal.pone.0020662)

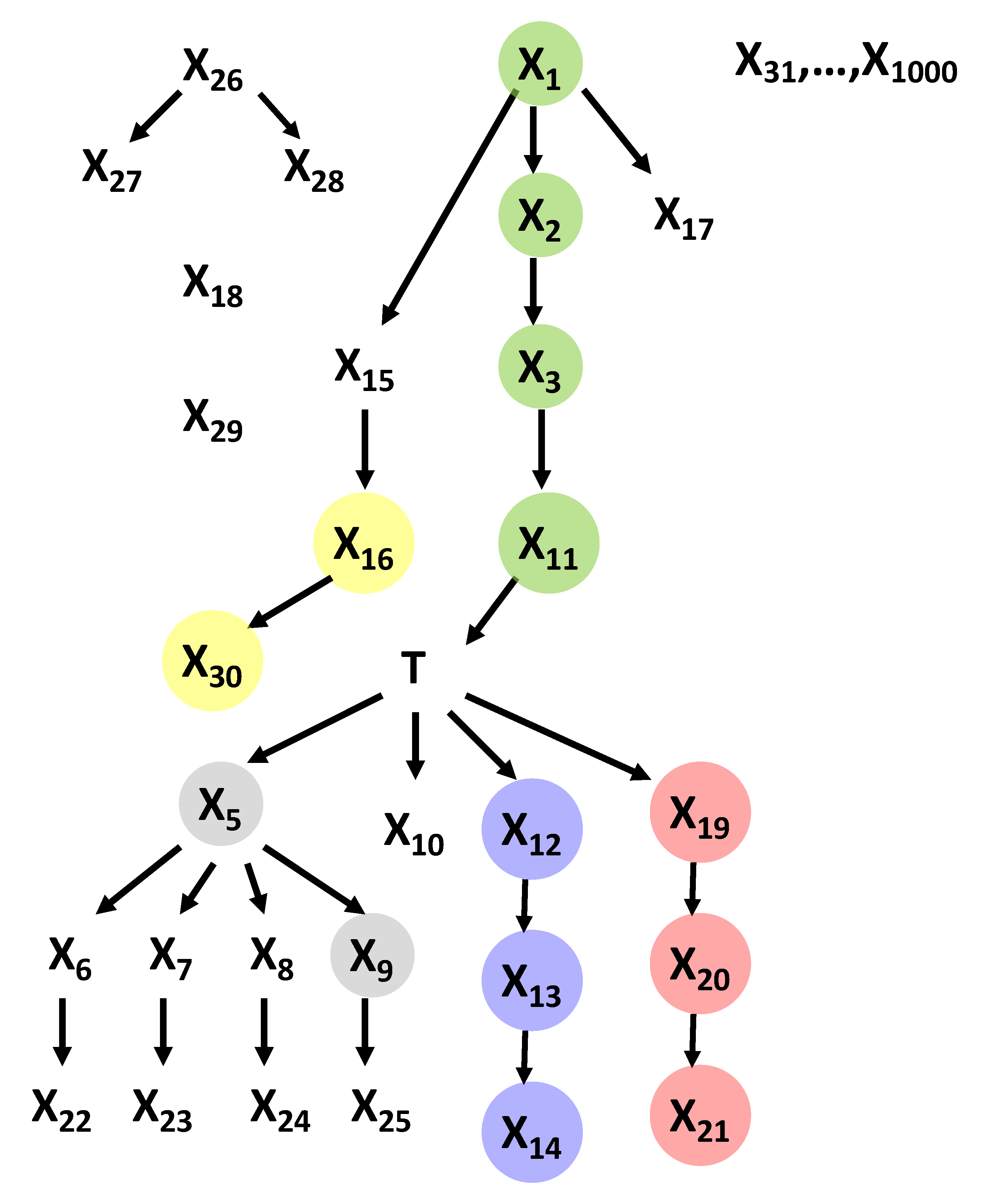

Supplement: Figure S1 — Data generating graph that was used for evaluation of methods for development of molecular signatures under the condition of signature multiplicity. There are 1,000 variables in the graph (999 genes and a phenotypic response variable T). Genes that contain exactly the same information about T are highlighted with the same color, e.g. genes X 12, X 13, and X 14 provide exactly the same information about T and are thus interchangeable for prediction of T. There are 72 distinct molecular signatures of the phenotype T (i.e., sets of non-redundant genes that carry maximal predictive information about the phenotype and render it statistically independent of all other genes). Each of these signatures carries equivalent information about the phenotype and spans over 5 genes: gene X 10 and one gene from each of the four subsets {X 1,X 2,X 3,X 11}, {X 5,X 9}, {X 12,X 13,X 14} and {X 19,X 20,X 21}. (TIF) [file pone.0020662.s001.tif]

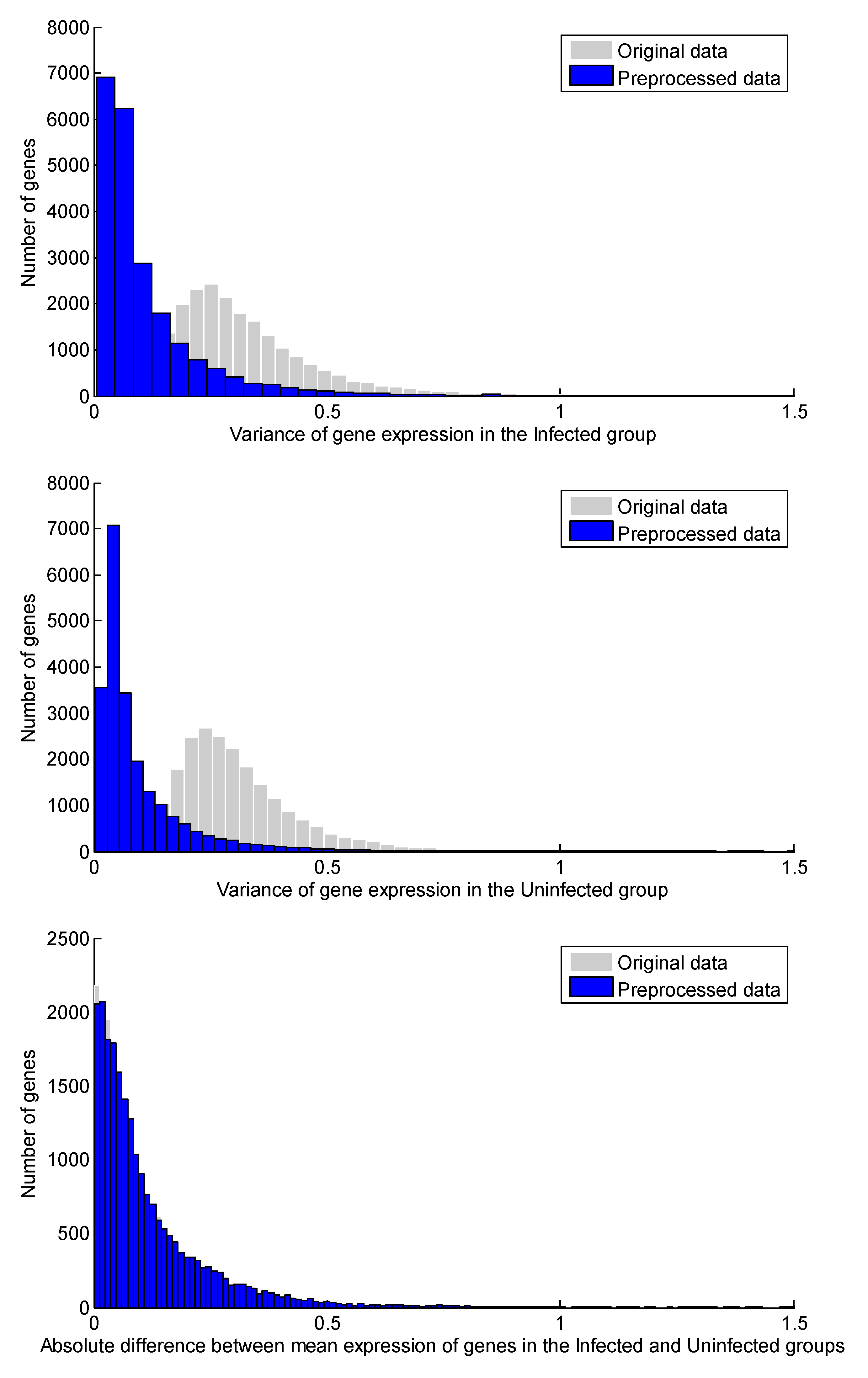

Supplement: Figure S2 — Distributions of variance in the infected and uninfected subjects (top two figures) and differences between means of their gene expression profiles (bottom) before and after preprocessing by the supplementary software of Zaas et al. [9] . The distribution of variance is shifted to the left (i.e., to smaller values) as a result of preprocessing, while the distribution of differences between means is largely unaffected. (TIF) [file pone.0020662.s002.tif]

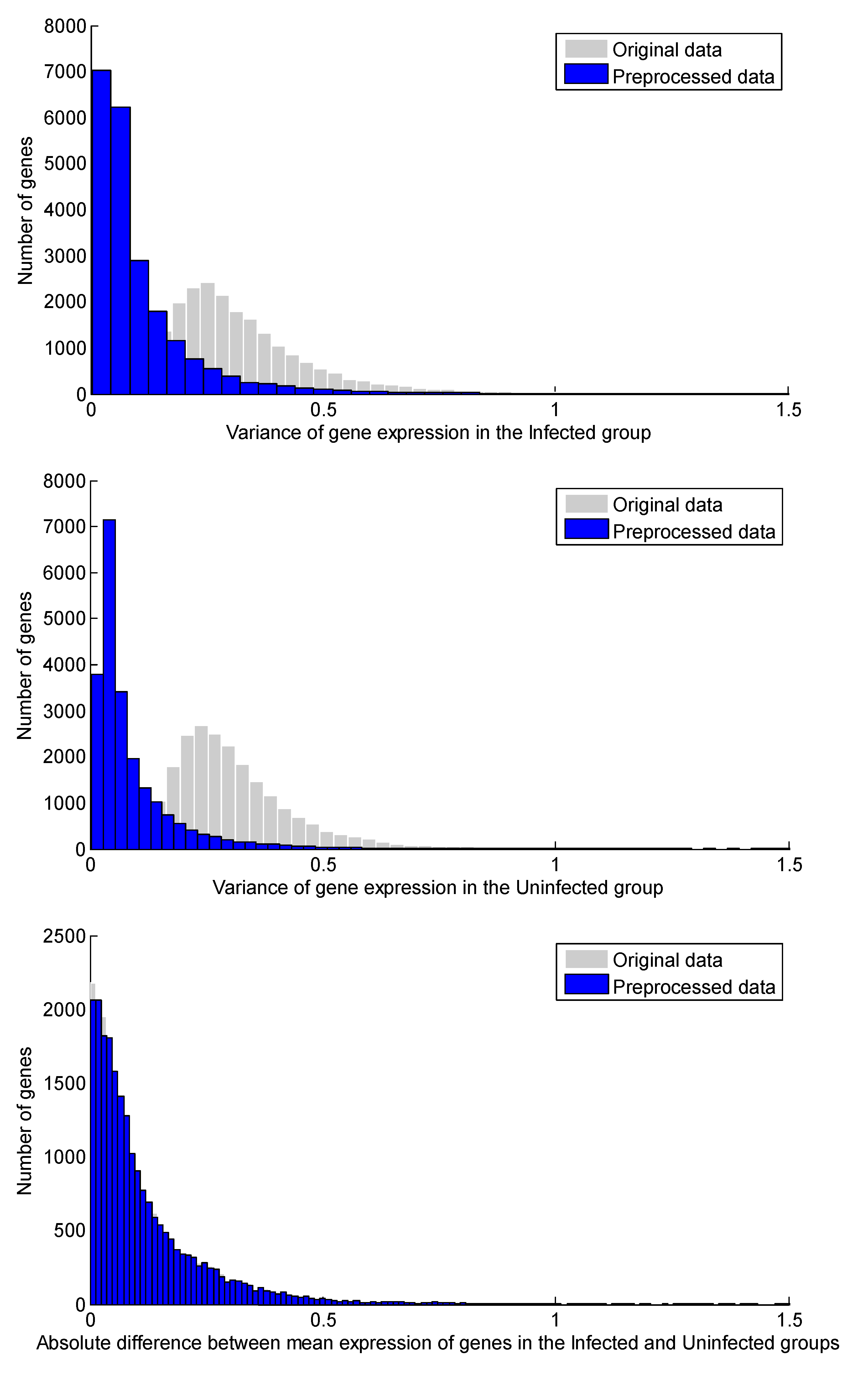

Supplement: Figure S3 — Distributions of variance in the infected and uninfected subjects (top two figures) and differences between means of their gene expression profiles (bottom) before and after preprocessing by ComBat [34] . The distribution of variance is shifted to the left (i.e., to smaller values) as a result of preprocessing, while the distribution of differences between means is largely unaffected. (TIF) [file pone.0020662.s003.tif]

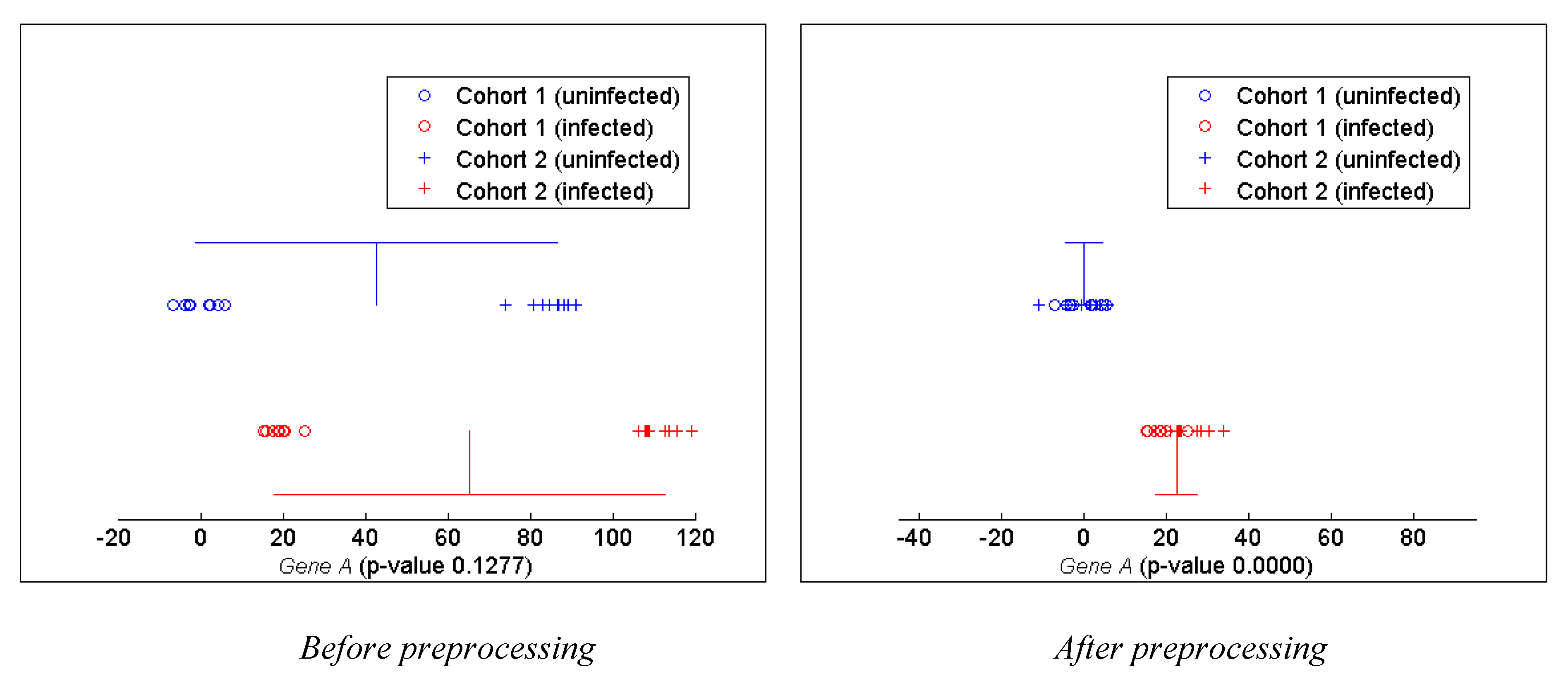

Supplement: Figure S4 — Effects of preprocessing method from the supplementary software of Zaas et al. [9] on simulated data. Gene expression profiles of the uninfected subjects are shown in blue staggered on top of the profiles of the infected subjects highlighted with red. The blue and red vertical line segments denote locations of the mean expression in the uninfected and infected groups, respectively. Likewise, blue and red horizontal line segments emanating in both directions from the means denote one standard deviation within the uninfected and infected groups, respectively. P-values produced by a two-sample t-test with unequal variances are shown in parenthesis. (TIF) [file pone.0020662.s004.tif]
