# Supplementary material for: Expanding the Understanding of Biases in Development of Clinical-Grade Molecular Signatures: A Case Study in Acute Respiratory Viral Infections
Source: PLoS One. 2011 Jun 1;6(6):e20662. doi: 10.1371/journal.pone.0020662 (PMC3105991; doi:10.1371/journal.pone.0020662)
Supplement: Table S1 — The complete list of genes participating in the 3,473 non-redundant and maximally predictive molecular signatures discovered by the TIE* algorithm in the data of Zaas et al. [9] for discriminating symptomatic from uninfected samples. Genes highlighted in bold are those that also comprised the 12-gene panviral signature developed by Statnikov et al. [10] by applying GLL on the entire set of samples. (PDF) [file pone.0020662.s005.pdf]

Table S1: The complete list of genes participating in the 3,473 non-redundant and maximally predictive molecular signatures discovered by the TIE* algorithm in the data of Zaas *et al.* [1] for discriminating symptomatic from uninfected samples.

Probe set ID	***Gene symbol***	***Gene name***	***Percentage of signatures participated in***
201065_s_at	***GTF2I***	general transcription factor IIi	72.93%
213674_x_at	***IGHD***	immunoglobulin heavy constant delta	72.53%
214511_x_at	***FCGR1B***	Fc fragment of IgG, high affinity Ib, receptor (CD64)	72.01%
207826_s_at	***ID3***	inhibitor of DNA binding 3, dominant negative helix-loop-helix protein	71.41%
213797_at	***RSAD2***	radical S-adenosyl methionine domain containing 2	70.66%
217418_x_at	***MS4A1***	membrane-spanning 4-domains, subfamily A, member 1	69.80%
219471_at	***C13orf18***	chromosome 13 open reading frame 18	69.39%
219112_at	***RAPGEF6***	Rap guanine nucleotide exchange factor (GEF) 6	63.29%
219073_s_at	***OSBPL10***	oxysterol binding protein-like 10	58.71%
219313_at	***GRAMD1C***	GRAM domain containing 1C	56.29%
204439_at	***IFI44L***	interferon-induced protein 44-like	42.38%
221234_s_at	*BACH2*	BTB and CNC homology 1, basic leucine zipper transcription factor 2	28.76%
216950_s_at	*FCGR1A, FCGR1C*	Fc fragment of IgG, high affinity Ia, receptor (CD64); Fc fragment of IgG, high affinity Ic, receptor (CD64)	27.99%
207431_s_at	***DEGS1***	degenerative spermatocyte homolog 1, lipid desaturase (Drosophila)	25.37%
205049_s_at	*CD79A*	CD79a molecule, immunoglobulin-associated alpha	23.73%
202723_s_at	*FOXO1*	forkhead box O1	22.40%
44790_s_at	*C13orf18*	chromosome 13 open reading frame 18	21.83%
203413_at	*NELL2*	NEL-like 2 (chicken)	19.95%
214059_at	*IFI44*	Interferon-induced protein 44	19.55%
206025_s_at	*TNFAIP6*	tumor necrosis factor, alpha-induced protein 6	19.00%
204747_at	*IFIT3*	interferon-induced protein with tetratricopeptide repeats 3	18.40%
218578_at	*CDC73*	cell division cycle 73, Paf1/RNA polymerase II complex component, homolog (S. cerevisiae)	15.38%
207180_s_at	*HTATIP2*	HIV-1 Tat interactive protein 2, 30kDa	12.44%
210448_s_at	*P2RX5*	purinergic receptor P2X, ligand-gated ion channel, 5	9.79%
208901_s_at	*TOP1*	topoisomerase (DNA) I	9.39%
210356_x_at	*MS4A1*	membrane-spanning 4-domains, subfamily A, member 1	9.39%
203153_at	*IFIT1*	interferon-induced protein with tetratricopeptide repeats 1	8.49%
205552_s_at	*OAS1*	2',5'-oligoadenylate synthetase 1, 40/46kDa	8.49%
217719_at	*EIF3L*	eukaryotic translation initiation factor 3, subunit L	8.12%
219452_at	*DPEP2*	dipeptidase 2	6.74%
202086_at	*MX1*	myxovirus (influenza virus) resistance 1, interferon-inducible protein p78 (mouse)	6.62%
209582_s_at	*CD200*	CD200 molecule	5.01%
208975_s_at	*KPNB1*	karyopherin (importin) beta 1	4.06%
208436_s_at	*IRF7*	interferon regulatory factor 7	3.80%
205483_s_at	*ISG15*	ISG15 ubiquitin-like modifier	2.07%
200689_x_at	*EEF1G*	eukaryotic translation elongation factor 1 gamma	1.99%
209185_s_at	*IRS2*	insulin receptor substrate 2	1.76%
215621_s_at	*IGHD*	immunoglobulin heavy constant delta	1.64%
210817_s_at	*CALCOCO2*	calcium binding and coiled-coil domain 2	1.47%
202748_at	*GBP2*	guanylate binding protein 2, interferon-inducible	1.41%
219394_at	*PGS1*	phosphatidylglycerophosphate synthase 1	1.07%

Genes highlighted in bold are those that also comprised the 12-gene panviral signature developed by Statnikov *et al.* [2] by applying GLL on the entire set of samples.

Continued on the next page

Table S1: Continued from the previous page.

Probe set ID	*Gene symbol*	*Gene name*	*Percentage of signatures participated in*
217986_s_at	*BAZ1A*	bromodomain adjacent to zinc finger domain, 1A	0.98%
204972_at	*OAS2*	2'-5'-oligoadenylate synthetase 2, 69/71kDa	0.66%
203038_at	*PTPRK*	protein tyrosine phosphatase, receptor type, K	0.37%
203939_at	*NT5E*	5'-nucleotidase, ecto (CD73)	0.35%
205660_at	*OASL*	2'-5'-oligoadenylate synthetase-like	0.32%
220179_at	*DPEP3*	dipeptidase 3	0.32%
202100_at	*RALB*	v-ral simian leukemia viral oncogene homolog B (ras related; GTP binding protein)	0.26%
204423_at	*MKLN1*	muskelin 1, intracellular mediator containing kelch motifs	0.26%
212821_at	*PLEKHG3*	pleckstrin homology domain containing, family G (with RhoGef domain) member 3	0.23%
201739_at	*SGK1*	serum/glucocorticoid regulated kinase 1	0.03%
204581_at	*CD22*	CD22 molecule	0.03%
206420_at	*IGSF6*	immunoglobulin superfamily, member 6	0.03%
209417_s_at	*IFI35*	interferon-induced protein 35	0.03%
218543_s_at	*PARP12*	poly (ADP-ribose) polymerase family, member 12	0.03%
219211_at	*USP18*	ubiquitin specific peptidase 18	0.03%
219364_at	*DHX58*	DEXH (Asp-Glu-X-His) box polypeptide 58	0.03%
219700_at	*PLXDC1*	plexin domain containing 1	0.03%
219863_at	*HERC5*	hect domain and RLD 5	0.03%
221239_s_at	*FCRL2*	Fc receptor-like 2	0.03%

References

1. Zaas AK, Chen M, Varkey J, Veldman T, Hero AO, III, Lucas J, Huang Y, Turner R, Gilbert A, Lambkin-Williams R, Oien NC, Nicholson B, Kingsmore S, Carin L, Woods CW, Ginsburg GS (2009) Gene expression signatures diagnose influenza and other symptomatic respiratory viral infections in humans. Cell Host Microbe 6: 207-217.

2. Statnikov A, McVoy L, Lytkin N, Aliferis CF (2010) Improving development of the molecular signature for diagnosis of acute respiratory viral infections. Cell Host Microbe 7: 100-101.
