# Supplementary material for: Expanding the Understanding of Biases in Development of Clinical-Grade Molecular Signatures: A Case Study in Acute Respiratory Viral Infections
Source: PLoS One. 2011 Jun 1;6(6):e20662. doi: 10.1371/journal.pone.0020662 (PMC3105991; doi:10.1371/journal.pone.0020662)
Supplement: Table S2 — The complete list of genes participating in the 2,922 non-redundant and maximally predictive molecular signatures discovered by TIE* in the data of Zaas et al. [11] for discriminating between Candidemia-infected samples and healthy controls. Genes highlighted in bold are those that also comprised a 14-gene signature developed by applying GLL in the same data. (PDF) [file pone.0020662.s006.pdf]

Table S2: The complete list of genes participating in the 2,922 non-redundant and maximally predictive molecular signatures discovered by TIE* in the data of Zaas *et al*. [1] for discriminating between Candidemia-infected samples and healthy controls.

Probe set ID	***Gene symbol***	***Gene title***	***Precentage of signatures participated in***
1420330_at	***Clec4e***	C-type lectin domain family 4, member e	100.00%
1424132_at	***Hras1***	Harvey rat sarcoma virus oncogene 1	100.00%
1427747_a_at	***Lcn2***	lipocalin 2	100.00%
1417293_at	***Hs6st1 /// LOC100047260***	heparan sulfate 6-O-sulfotransferase 1 /// similar to heparan sulfate 6-sulfotransferase 1	73.51%
1426739_at	***Donson***	downstream neighbor of SON	72.25%
1427011_a_at	***Lancl1***	LanC (bacterial lantibiotic synthetase component C)-like 1	71.42%
1427925_at	***Stx17***	syntaxin 17	71.36%
1452197_at	***Smc4***	structural maintenance of chromosomes 4	71.01%
1428357_at	***2610019F03Rik***	RIKEN cDNA 2610019F03 gene	70.71%
1416378_at	***Pnkp***	polynucleotide kinase 3'- phosphatase	69.75%
1452504_s_at	***Ctbs***	chitobiase, di-N-acetyl-	66.80%
1417933_at	***Igfbp6***	insulin-like growth factor binding protein 6	56.71%
1422438_at	***Ephx1***	epoxide hydrolase 1, microsomal	52.05%
1416524_at	***Spop***	speckle-type POZ protein	46.68%
1418012_at	*Sh3glb1*	SH3-domain GRB2-like B1 (endophilin)	41.75%
1424754_at	*Ms4a7*	membrane-spanning 4-domains, subfamily A, member 7	33.85%
1427030_at	*Ccdc52*	coiled-coil domain containing 52	33.57%
1429800_at	*9130221H12Rik /// Zfp526*	RIKEN cDNA 9130221H12 gene /// zinc finger protein 526	32.58%
1416988_at	*Msh2*	mutS homolog 2 (E. coli)	32.27%
1451092_a_at	*Rangap1*	RAN GTPase activating protein 1	29.23%
1417164_at	*Dusp10*	dual specificity phosphatase 10	20.91%
1423466_at	*Ccr7*	chemokine (C-C motif) receptor 7	20.84%
1454722_at	*Pten*	phosphatase and tensin homolog	18.24%
1448361_at	*Ttc3*	tetratricopeptide repeat domain 3	16.91%
1460551_at	*Ran*	RAN, member RAS oncogene family	16.77%
1416226_at	*Arpc1b*	actin related protein 2/3 complex, subunit 1B	14.82%
1449945_at	*Ppargc1b*	peroxisome proliferative activated receptor, gamma, coactivator 1 beta	11.33%
1416359_at	*Snx18*	sorting nexin 18	7.91%
1448129_at	*Arpc5*	actin related protein 2/3 complex, subunit 5	7.73%
1424369_at	*Psmf1*	proteasome (prosome, macropain) inhibitor subunit 1	5.30%
1423371_at	*Pole4*	polymerase (DNA-directed), epsilon 4 (p12 subunit)	5.13%
1419833_s_at	*Arap3*	ArfGAP with RhoGAP domain, ankyrin repeat and PH domain 3	3.87%
1418396_at	*Gpsm3*	G-protein signalling modulator 3 (AGS3-like, C. elegans)	2.67%
1423539_at	*Pms2*	postmeiotic segregation increased 2 (S. cerevisiae)	2.67%
1416759_at	*Mical1*	microtubule associated monoxygenase, calponin and LIM domain containing 1	2.22%
1422637_at	*Rassf5*	Ras association (RalGDS/AF-6) domain family member 5	2.22%
1434998_at	*Iqgap1*	IQ motif containing GTPase activating protein 1	2.22%
1416629_at	*Slc1a5*	solute carrier family 1 (neutral amino acid transporter), member 5	1.81%
1460436_at	*Ndst1*	N-deacetylase/N-sulfotransferase (heparan glucosaminyl) 1	1.27%
1433507_a_at	*Hmgn2*	high mobility group nucleosomal binding domain 2	1.10%

Genes highlighted in bold are those that also comprised a 14-gene signature developed by applying GLL in the same data.

Continued on the next page

Table S2: Continued from the previous page.

Probe set ID	*Gene symbol*	*Gene title*	*Precentage of signatures participated in*
1418634_at	*Notch1*	Notch gene homolog 1 (Drosophila)	1.06%
1449883_at	*Fxyd2*	FXYD domain-containing ion transport regulator 2	1.06%
1436180_at	*Dnajc5*	DnaJ (Hsp40) homolog, subfamily C, member 5	0.62%
1419810_x_at	*Arhgap9*	Rho GTPase activating protein 9	0.41%
1421038_a_at	*Kcnn4*	potassium intermediate/small conductance calcium-activated channel, subfamily N, member 4	0.41%
1424526_a_at	*Tgds*	TDP-glucose 4,6-dehydratase	0.41%
1436838_x_at	*Cotl1*	coactosin-like 1 (Dictyostelium)	0.41%
1448407_at	*4632428N05Rik*	RIKEN cDNA 4632428N05 gene	0.41%
1449111_a_at	*Grb2*	growth factor receptor bound protein 2	0.41%
1434561_at	*Asxl1*	additional sex combs like 1 (Drosophila)	0.38%
1426212_s_at	*Tmem161a*	transmembrane protein 161A	0.34%
1437773_x_at	*Ddx17*	DEAD (Asp-Glu-Ala-Asp) box polypeptide 17	0.34%
1419731_at	*Cyp2b19*	cytochrome P450, family 2, subfamily b, polypeptide 19	0.31%
1428736_at	*Gramd3*	GRAM domain containing 3	0.31%
1451125_at	*Paip2b*	poly(A) binding protein interacting protein 2B	0.31%
1417171_at	*Itk*	IL2-inducible T-cell kinase	0.03%
1417597_at	*Cd28*	CD28 antigen	0.03%
1418468_at	*Anxa11 /// Gm2260 /// Gm2274*	annexin A11 /// predicted gene 2260 /// predicted gene 2274	0.03%
1419193_a_at	*Gmfg*	glia maturation factor, gamma	0.03%
1423150_at	*Scg5*	secretogranin V	0.03%
1423897_at	*Rnf187*	ring finger protein 187	0.03%
1424168_a_at	*Capzb*	capping protein (actin filament) muscle Z-line, beta	0.03%
1427108_at	*9530068E07Rik*	RIKEN cDNA 9530068E07 gene	0.03%
1451181_at	*Tmem121*	transmembrane protein 121	0.03%
1460555_at	*Fam65b*	family with sequence similarity 65, member B	0.03%

References

1. Zaas AK, Aziz H, Lucas J, Perfect JR, Ginsburg GS (2010) Blood gene expression signatures predict invasive candidiasis. Sci Transl Med 2: 21ra17.
