# Supplementary material for: Expanding the Understanding of Biases in Development of Clinical-Grade Molecular Signatures: A Case Study in Acute Respiratory Viral Infections
Source: PLoS One. 2011 Jun 1;6(6):e20662. doi: 10.1371/journal.pone.0020662 (PMC3105991; doi:10.1371/journal.pone.0020662)
Supplement: Text S1 — An illustration of the effects of a preprocessing procedure from the supplementary software of Zaas et al. [9]. (PDF) [file pone.0020662.s009.pdf]

Supplementary Text S1: An illustration of the effects of a preprocessing procedure from the supplementary software of Zaas *et al.* [1]

The original data of Zaas *et al.* [1] (i.e., raw probe data after RMA normalization [2,3]) is particularly suitable for an illustration of the effects of a preprocessing procedure implemented in their supplementary software. This dataset exhibits significant differences between the unexposed (uninfected and healthy) subjects from the three viral cohorts. Figure (a) shows gene expression profiles of the unexposed subjects in the space of the first two principal components of the original data. The three clearly distinct groups of subjects marked with diamonds, crosses and triangles in Figure (a) correspond exactly to the unexposed subjects in HRV, RSV and influenza cohorts, respectively. One can only hypothesize about the causes of differences between the unexposed subjects, as these differences were not discussed in the primary study [1]. When applied to this data, the preprocessing procedure fused gene expression profiles of the unexposed subjects into a single group (see Figure (b)) resulting in reduction of variance of gene expression across these subjects. The profiles of the infected subjects were similarly affected.

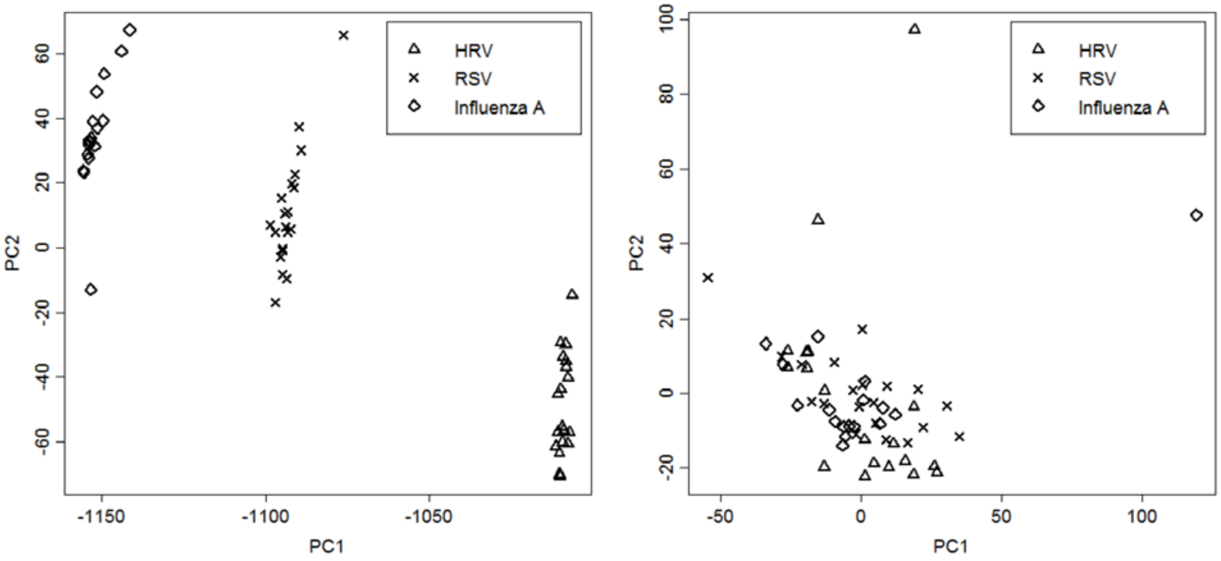

(a) (b)

Figure: An illustration of a preprocessing method from the supplementary software of Zaas *et al.* (a) Plot of gene expression profiles of the unexposed subjects in the space of the first two principal components using *original data* from [1]. (b) Plot of gene expression profiles of the unexposed subjects in the space of the first two principal components of the original data after preprocessing using the supplementary software of Zaas *et al*. [1]

References

1. Zaas AK, Chen M, Varkey J, Veldman T, Hero AO, III, Lucas J, Huang Y, Turner R, Gilbert A, Lambkin-Williams R, Oien NC, Nicholson B, Kingsmore S, Carin L, Woods CW, Ginsburg GS (2009) Gene expression signatures diagnose influenza and other symptomatic respiratory viral infections in humans. Cell Host Microbe 6: 207-217.

2. Irizarry RA, Hobbs B, Collin F, Beazer-Barclay YD, Antonellis KJ, Scherf U, Speed TP (2003) Exploration, normalization, and summaries of high density oligonucleotide array probe level data. Biostatistics 4: 249-264.

3. Irizarry RA, Wu Z, Jaffee HA (2006) Comparison of Affymetrix GeneChip expression measures. Bioinformatics 22: 789-794.
